# EZH2 promotes colorectal cancer stem-like cell expansion by activating p21^cip1^-Wnt/β-catenin signaling

**DOI:** 10.18632/oncotarget.9236

**Published:** 2016-05-09

**Authors:** Jian-Fang Chen, Xi Luo, Li-Sha Xiang, Hong-Tao Li, Lin Zha, Ni Li, Jian-Ming He, Gan-Feng Xie, Xiong Xie, Hou-Jie Liang

**Affiliations:** ^1^ Department of Oncology and Southwest Cancer Center, Southwest Hospital, Third Military Medical University, Chongqing, China; ^2^ State Key Laboratory of Biotherapy and Cancer Center, West China Hospital, Sichuan University, Sichuan, China

**Keywords:** EZH2, cancer stem-like cell, colorectal cancer, cell cycle, p21^cip1^

## Abstract

Because colorectal cancer (CRC) stem-like cells (CCS-like cells) contribute to poor patient prognosis, these cells are a potential target for CRC therapy. However, the mechanism underlying the maintenance of CCS-like cell properties remains unclear. Here, we found that patients with advanced stage CRC expressed high levels of polycomb group protein enhancer of zeste homologue 2 (EZH2). High expression of EZH2 in tumor tissues correlated with poor patient prognosis. Conversely, silencing EZH2 reduced CRC cell proliferation. Surprisingly, EZH2 was more highly expressed in the CCS-like cell subpopulation than in the non-CCS-like cell subpopulation. EZH2 knockdown significantly reduced the CD133^+^/CD44^+^ subpopulation, suppressed mammosphere formation, and decreased the expression of self-renewal-related genes and strongly impaired tumor-initiating capacity in a re-implantation mouse model. Gene expression data from 433 human CRC specimens from TCGA database and *in vitro* results revealed that EZH2 helped maintain CCS-like cell properties by activating the Wnt/β-catenin pathway. We further revealed that p21^cip1^–mediated arrest of the cell cycle at G1/S phase is required for EZH2 activation of the Wnt/β-catenin pathway. Moreover, the specific EZH2 inhibitor EPZ-6438, a clinical trial drug, prevented CRC progression. Collectively, these findings revealed EZH2 maintaining CCS-like cell characteristics by arresting the cell cycle at the G1/S phase. These results indicate a new approach to CRC therapy.

## INTRODUCTION

Colorectal cancer (CRC) is one of the most commonly diagnosed cancers, and 1.23 million patients are diagnosed with CRC each year worldwide [1]. Despite recent advances in chemotherapies that have improved survival rates, patients with late-stage disease still have a poor prognosis, and the overall mortality rate of CRC is approximately 40% [2–5]. Therefore, new effective therapeutic strategies are urgently needed [6].

CRC stem-like cells (CCS-like cells) have recently attracted increased attention due to their contribution to the poor prognosis of cancer patients [7–11]. Emerging evidence has suggested that cancer stem-like cells (CS-like cells) in the primary tumor possess the abilities to self-renew and differentiate [12]. CS-like cells promote tumorigenesis, recurrence and metastasis [13]. The 5-year survival rate was significantly higher in cancer cases with a low percentage of CCS-like cells (below 5%) than in those with a higher percentage of CCS-like cells [14]. Dysregulation of developmental pathway members, including Wnt, Hedgehog, Notch, and transforming growth factor-beta/bone morphogenetic protein, contributes to the maintenance of CS-like cell properties [15, 16]. Aberrant Wnt signaling pathway activation predicts poor CRC patient prognosis [17]. Wnt cascade activity is a critical regulator maintaining CCS-like cell properties [18]. The mechanism by which Wnt/β-catenin maintains CS-like cell properties has been identified [19–21]. However, the critical activator of the Wnt signaling pathway in CCS-like cells is largely unknown.

Epigenetic regulation by polycomb proteins is an essential factor in the poor prognosis of cancer patients [22, 23]. High expression of polycomb group protein enhancer of zeste homologue 2 (EZH2), a key component of the polycomb PRC2 complex, links to aggressive cancer progression [24, 25]. EZH2 has been shown to be overexpressed in cancers [22]. Moreover, EZH2 expression is associated with a high proliferation rate and aggressive tumor subtypes of cancer [26]. However, the function of EZH2 in maintaining CCS-like cell properties is unknown.

Recent studies have shown that cell cycle inhibitor gene expression is involved in attenuating CS-like cell capabilities [27–29]. Interestingly, decreasing EZH2 expression has been shown to arrest Temozolomide-resistant glioblastoma cells at the G1/S phase [30, 31]. Additionally, genetic reducing EZH2 expression has been shown to increase the expression of the cell cycle inhibitor p21^cip1^ (also referred to as p21^waf1^) in embryonal rhabdomyosarcoma [32–35]. However, the role of p21^cip1^ in reducing the CS-like cell population is controversial. Studies have suggested that p21^cip1^ expression promoted tumor regression and poor prognosis in 112 cancer patients [36, 37], but other studies reported that p21^cip1^ expression reduces stem cell properties [38, 39]. Notably, increased p21 signaling in CRC inactivates the Wnt/β-catenin pathway [40]. Thus, it is important to study the function of p21^cip1^ in the activation of the Wnt/β-catenin pathway by EZH2.

In this study, we demonstrated that EZH2 promotes CS-like cell properties in CRC and further clarified the mechanism by which EZH2 expression activates Wnt/β-catenin signaling in CRC. Moreover, we revealed that pharmacologically disrupting EZH2 using the clinical trial drug EPZ-6438 prevented CRC progression.

## RESULTS

### EZH2 acted as a predictive marker for CRC prognosis

**Figure 1:**
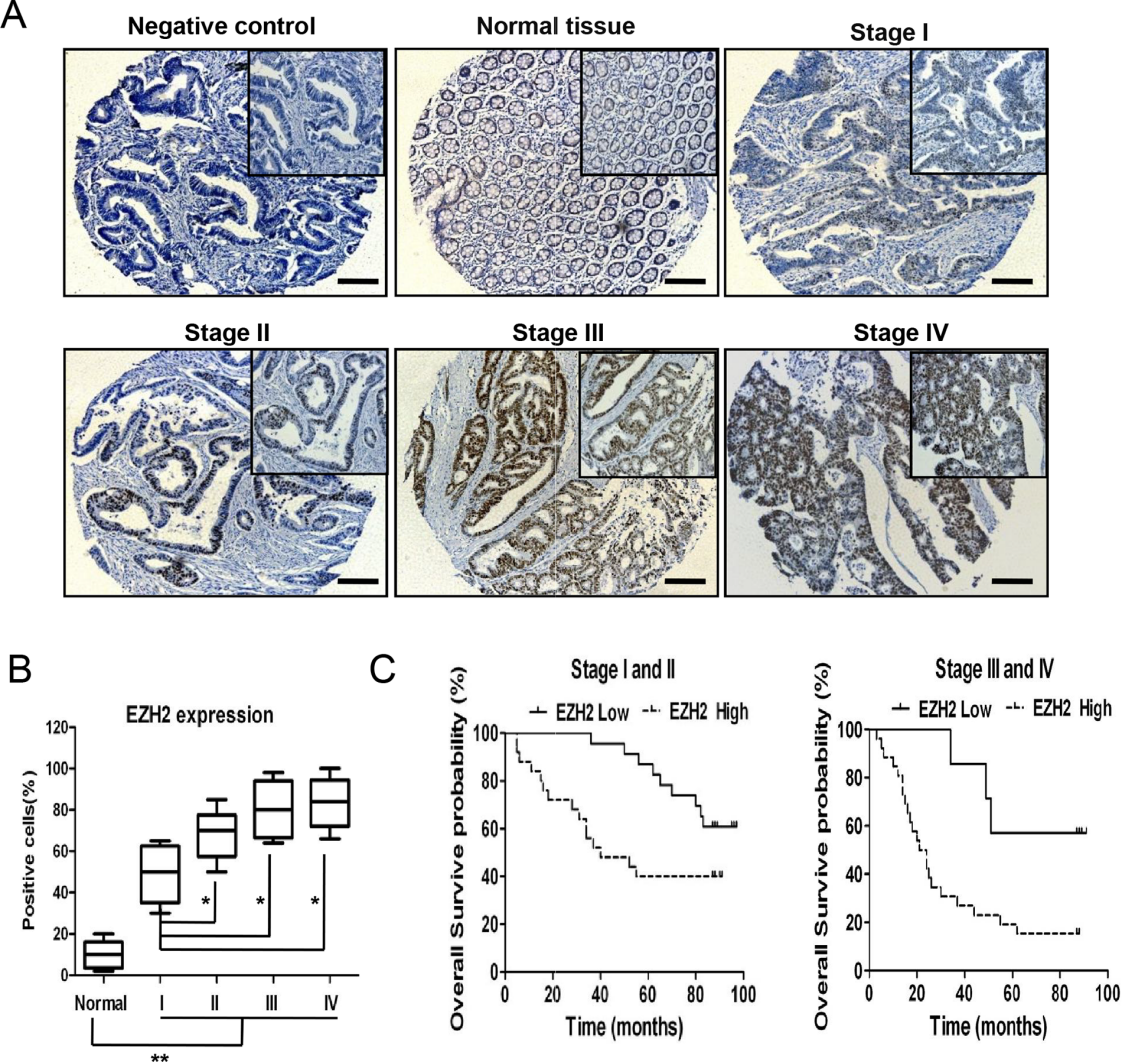
EZH2 acts as a predictive marker for CRC prognosis **A.** EZH2 immunohistochemistry of a tissue chip containing normal human colorectal tissues and CRC tissues at different pathological stages. **B.** The EZH2 expression levels were compared between the normal colorectal tissues and the CRC tissues at different pathological stages. The IRS was determined as described in the methods. **C.** Statistical analysis of the correlation between the EZH2 expression level and the overall survival of CRC patients (stages I and II, n = 48; stages III and IV, n = 33). The IRS was determined as described in the methods (*p<0.05 and **p<0.01, two-tailed Student's t-tests; the error bars represent the means ± S.D.).

To identify the relationship between EZH2 expression and CRC patient prognosis, we analyzed 81 CRC specimens via immunohistochemical staining (Table 1). EZH2 expression was substantially higher in CRC samples than in normal colon tissue samples (p<0.01), and EZH2 expression gradually increased with increasing pathological stage (from stage I to stage IV, p<0.05) (Figure 1A-1B). Notably, according to Kaplan-Meier analysis, patients with high EZH2 expression experienced poorer overall survival (OS) than those with low EZH2 expression in early stage (I and II) (Figure 1C left panel) and advanced stage (III and IV) tumors (Figure 1C right panel). These data suggested that EZH2 acts as a predictive marker for CRC prognosis.

**Table 1:** The correlation between EZH2 expression levels and clinicopathological features in colorectal cancer

Variables	Number of Patients	EZH2 expression index (IRS)			Fisher's (*P*)
		<3	3-7	>7	
Age (years)					0.617
<60	15	0 (0.0)	6 (40.0)	9(60.0)	
≥60	66	4 (6.1)	20 (30.3)	42 (63.6)	
Gender					0.291
Male	41	1 (2.4)	16 (39.0)	24 (58.5)	
Female	40	3 (7.5)	10 (25.0)	27 (67.5)	
Differentiation					0.459
well	5	0 (0.0)	2 (40.0)	3 (60.0)	
moderate	47	4 (8.5)	16 (34.0)	27 (57.4)	
poor	29	0 (0.0)	8 (27.6)	21 (72.4)	
Tumor status					0.160
T1	3	0 (0.0)	0 (0.0)	3 (100.0)	
T2	6	0 (0.0)	2 (33.3)	4 (66.7)	
T3	63	4 (6.3)	24 (38.1)	35 (55.6)	
T4	9	0 (0.0)	0 (0.0)	9 (100.0)	
Nodal status					0.071
N0	49	3 (6.1)	20 (40.8)	26 (53.1)	
N1-2	32	1 (3.1)	6 (18.8)	25 (78.1)	
Metastasis status					0.591
M0	79	4 (5.1)	26 (32.9)	49(62.0)	
M1	2	0 (0.0)	0 (0.0)	2 (100.0)	
Clinical stage					0.048
I+II	48	3 (6.3)	20 (41.7)	25 (52.1)	
III+IV	33	1 (3.0)	6 (18.2)	26 (78.8)	

### EZH2 promoted CRC cell proliferation and clonogenicity

**Figure 2:**
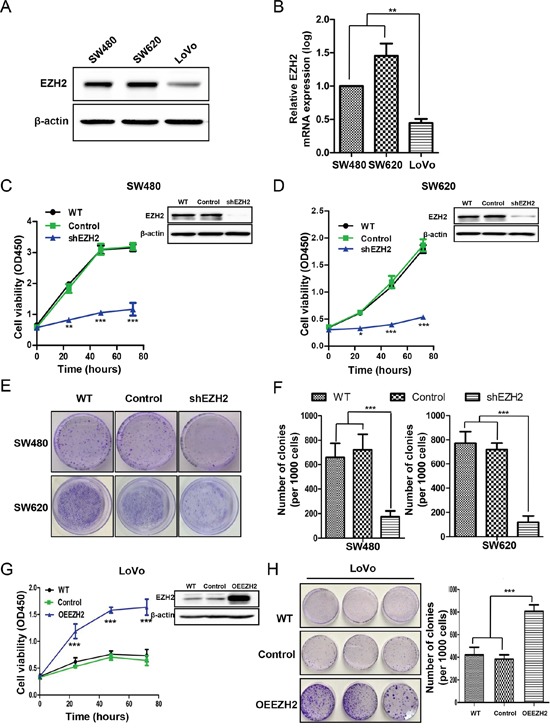
EZH2 promoted CRC cell proliferation and clonogenicity **A.** EZH2 protein expression in different CRC cell lines (SW480, SW620 and LoVo) was assessed by western blot. Representative results of EZH2 expression from three independent experiments are presented. **B.** The EZH2 mRNA levels in different CRC cell lines were assessed by qRT-PCR. **C.** EZH2 expression in WT cells, scramble shRNA-transfected cells (Control) and EZH2 shRNA-transfected cells was evaluated by western blot (right top corner), and cell viability was assessed via CCK-8 assays at the indicated time points in the SW480 and **D.** SW620 cell lines. **E.** Representative images from colony formation assays of WT, control-transfected and shEZH2-transfected SW480 and SW620 cells. **F.** Statistical analysis of the number of colonies from three independent experiments. **G.** EZH2 expression in WT, scramble control-transfected and OEEZH2 LoVo cells was evaluated by western blot (right top corner), and cell viability was assessed via CCK-8 assays at the indicated time points. **H.** Colony formation assays were performed on WT, scramble control-transfected and OEEZH2 cells, and the number of colonies from three independent experiments was statistically analyzed (*p<0.05, **p<0.01 and ***p<0.001, two-tailed Student's t-tests; the error bars represent the means ± S.D.).

To explore the potential role of EZH2 in CRC progression, we first investigated EZH2 expression in CRC cell lines (SW480, SW620 and LoVo) with varying proliferative abilities [41] by western blot and qRT-PCR analyses. EZH2 expression was higher in SW480 and SW620 cells, which have higher proliferation capacities than LoVo cells (Figure 2A). Additionally, the EZH2 transcription levels were higher in SW480 and SW620 cells than in LoVo cells (Figure 2B). We knocked down EZH2 expression using short hairpin RNA (shRNA) in SW480 and SW620 cells and performed western blotting to validate the knockdown efficiency. As depicted in the right top panels of Figure 2C and Figure 2D, EZH2 expression in SW480 and SW620 cells was nearly abolished by shEZH2 transfection. We determined cell viability at the indicated time points using a CCK-8 assay. Compared to the wild type (WT) and control groups, the shEZH2-transfected group displayed significantly decreased viability of SW480 cells (Figure 2C) and SW620 cells (Figure 2D). Moreover, we confirmed a significant time-dependent inhibition of SW480 and SW620 cell proliferation by shEZH2 transfection using a cell counting assay (Figure S1A-S1B). To further verify the inhibitory effect of shEZH2 on CRC cell growth, we performed colony formation assays after shRNA transfection (Figure 2E). Silencing EZH2 significantly reduced the number of colonies from 660.3 ± 114.7 of the WT cells and 723.3 ± 126.5 of the control cells to 174.0 ± 48.1 of the shEZH2-transfected cells in SW480 cells (Figure 2F left panel) and from 772.6 ± 95.3 of the WT cells and 720.7 ± 53.0 of the control cells to 119.7 ± 51.8 of the shEZH2-transfected cells in SW620 cells (Figure 2F right panel). Conversely, we overexpressed EZH2 in LoVo cells, which express low level of EZH2 (Figure 2G right panel). The CCK-8 assay showed that EZH2-overexpressing (OEEZH2) cells possessed greater viability than WT cells and scramble-transfected control cells (Control) (Figure 2G left panel). The colony number was significantly higher in OEEZH2 cells than in WT and control cells (Figure 2H). These results demonstrated that EZH2 promotes CRC cell proliferation.

### EZH2 expression increased in CCS-like cell subpopulation

**Figure 3:**
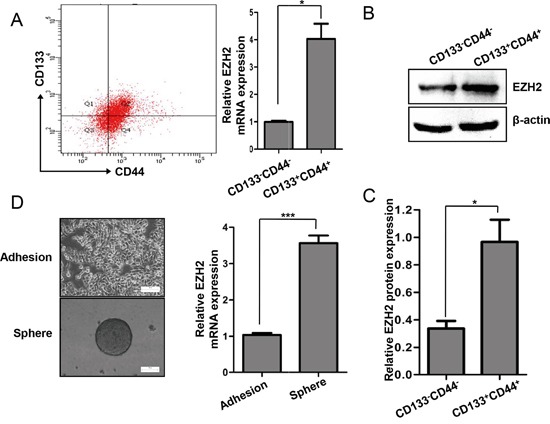
EZH2 expression was increased in the CCS-like cell subpopulation **A.** Representative flow cytometry assay results for CD133 and CD44 expression in SW480 cells are shown in the left panel. The relative mRNA expression levels of EZH2 in the CD133^−^/CD44^−^ and CD133^+^/CD44^+^ populations of SW480 cells are shown in the right panel. **B.** The protein expression levels of EZH2 in the CD133^−^/CD44^−^ and CD133^+^/CD44^+^ populations of SW480 cells were analyzed by western blot. **C.** The relative protein expression levels of EZH2 in the CD133^−^/CD44^−^ and CD133^+^/CD44^+^ populations of SW480 cells was statistically analyzed based on the results of western blots from three independent experiments. **D.** EZH2 mRNA expression in adherent cells and mammosphere cells was analyzed by qRT-PCR. Representative images of the cells are presented. The data were expressed as the means ± S.D. of three independent experiments (*p<0.05 and ***p<0.001, two-tailed Student's t-tests; the error bars represent the means ± S.D.).

The CCS-like cell subpopulation has been reported to play a critical role in tumor growth and progression, both of which contribute to poor prognosis [7, 8, 10, 11, 42]. Given that EZH2 expression is increased in advanced carcinomas and that EZH2 expression is essential for CRC cell growth, we hypothesized that EZH2 is differentially expressed between CS-like cells and non-CS-like cells. Previous studies have suggested that undifferentiated tumorigenic CD133^+^ and CD44^+^ cells possessed CCS-like cell properties [43, 44]. Here, we sorted the CD133^+^/CD44^+^ and CD133^−^/CD44^−^ subpopulations by flow cytometry (Figure 3A left panel). The EZH2 mRNA level was significantly increased in the CD133^+^/CD44^+^ subpopulation compared to the CD133^−^/CD44^−^ subpopulation (p<0.05) (Figure 3A right panel). Moreover, the EZH2 protein levels were higher in CD133^+^/CD44^+^ cells than in CD133^−^/CD44^−^ cells (Figure 3B-3C). We next performed mammosphere formation assay to test the self-renewal capacity of cells, which is a pivotal property of CS-like cells *in vitro* [45, 46]. We compared EZH2 mRNA expression between adherent SW480 cells and SW480 mammospheres. Indeed, the EZH2 mRNA level was >2.0-fold higher in mammosphere cells compared to adherent cells (Figure 3D). These findings demonstrated that EZH2 expression is higher in CCS-like cells than in non-CCS-like cells.

### EZH2 was indispensable for CCS-like cell maintenance *in vitro*

**Figure 4:**
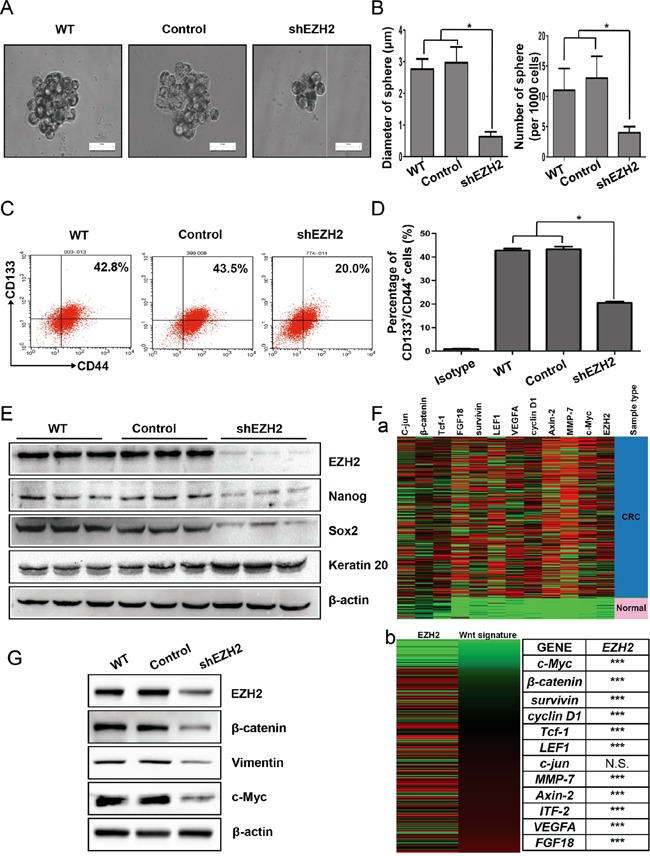
EZH2 was indispensable for CCS-like cell maintenance *in vitro* **A.** WT, scramble shRNA-transfected (Control) and shEZH2-transfected SW480 cells were plated under low-serum non-adherent culture conditions. Images were obtained via microscopy at 10× magnification, and the results shown are representative of all mammospheres formed (scale bar = 100 μm). **B.** The number and size of SW480 cell spheres were analyzed. The data are presented as the means ± S.D. of three independent experiments. **C.** The CD133^+^/CD44^+^ population of SW480 cells with or without EZH2 knockdown was analyzed by flow cytometry. **D.** The statistical results of three independent experiments are presented. **E.** After EZH2 knockdown, the expression of EZH2, Nanog, Sox2, and Keratin 20 was examined by western blot. **F.** a. The relative mRNA expression levels of EZH2 and Wnt target genes from TCGA colon and rectal adenocarcinoma (COADREAD) database of 433 CRC patient specimens were analyzed via RNAseq (IlluminaHiSeq). b. The expression of a Wnt/β–catenin signature significantly positively correlated with EZH2 expression (***p<0.0001, N.S., no significant correlation, Pearson's correlation test). **G.** The expression levels of β–catenin, vimentin and c-Myc were detected by western blot after EZH2 knockdown in SW480 cells (*p<0.05, two-tailed Student's t-tests; the error bars represent the means ± S.D.).

Because CCS-like cells preferentially expressed EZH2, we speculated that EZH2 expression plays an essential role in maintaining CCS-like cell properties. After shEZH2 transfection, we cultured SW480 cells in serum-free sphere formation medium (Figure 4A). EZH2-silenced cells exhibited significantly reduced mammosphere growth, as analyzed by sphere size (~75%, p<0.05) (Figure 4B left panel), and fewer mammospheres (~50%, p<0.05) (Figure 4B right panel) compared to WT or control cells.

To further establish whether EZH2 knockdown reduced the CCS-like cell population, we performed flow cytometry to analyze the percentage of cells in the CD133^+^/CD44^+^ subpopulation. As shown in Figure 4C, ~40% of WT and control SW480 cells were members of the CD133^+^/CD44^+^ subpopulation. Upon EZH2 knockdown, the CD133^+^/CD44^+^ subpopulation of SW480 cells dramatically decreased from 42.8 ± 0.8% of the WT cells and 43.3 ± 1.1% of the control cells to 20.5 ± 0.5% of the shEZH2-transfected cells (p<0.05) (Figure 4D). Additionally, the expression of self-renewal genes such as Nanog and Sox2 were decreased in shEZH2-transfected cells compared to WT and control SW480 cells (Figure 4E). In contrast, the expression of the differentiation marker Keratin 20 [47] was markedly increased by EZH2 knockdown (Figure 4E).

The Wnt/β-catenin pathway is essential for CCS-like cell maintenance [18]. Therefore, gene expression data from 433 human CRC specimens in The Cancer Genome Atlas (TCGA) database were used to analyze the mRNA expression levels of EZH2 relative to the levels of randomly chosen target genes in the Wnt/β-catenin pathway (http://web.stanford.edu/group/nusselab/cgi-bin/wnt/target_genes) (Figure 4F-a). The expression of a Wnt signature, based on the combined mRNA expression of all 12 Wnt/β-catenin pathway target genes, positively correlated with EZH2 mRNA expression (Figure 4F-b left panel). Statistical analysis revealed that EZH2 expression significantly positively correlated with 11 of the 12 Wnt/β-catenin pathway target genes (Figure 4F-b right panel). We therefore examined whether EZH2 participates in Wnt/β-catenin pathway activation in CRC cell lines *in vitro*. Compared to WT and control cells, EZH2-silenced cells displayed decreased β-catenin expression (Figure 4G). Moreover, EZH2 knockdown decreased the expression of c-Myc and vimentin (Figure 4G), which are known downstream target genes of the Wnt/β-catenin pathway [48]. We observed similar results in SW620 cells (Figure S2).

Conversely, after overexpressing the EZH2 protein in LoVo cells, sphere formation was examined, and represented images are presented in Figure S3A. The diameter and number of spheres in the OEEZH2 group were greater than those in the WT and scramble sequence-transfected (control) groups (Figure S3B). Although LoVo cells rarely expressed CD133^+^ [49], the CD44^+^ cell population was significantly increased in OEEZH2 cells compared with WT and control cells (Figure S3C-S3D). Additionally, the expression levels of Nanog and Sox2 were increased, and Keratin 20 were decreased in OEEZH2 cells compared to WT and control LoVo cells (Figure S3E). The β-catenin, c-Myc and vimentin levels were increased following EZH2 overexpression (Figure S3F). These results showed that the Wnt/β-catenin pathway was activated by EZH2. Collectively, these findings demonstrated that EZH2 is indispensable for activating Wnt/β-catenin signaling pathway to maintain CCS-like cell properties.

### EZH2 knockdown suppressed tumorigenesis and tumor-initiating capacity *in vivo*

**Figure 5:**
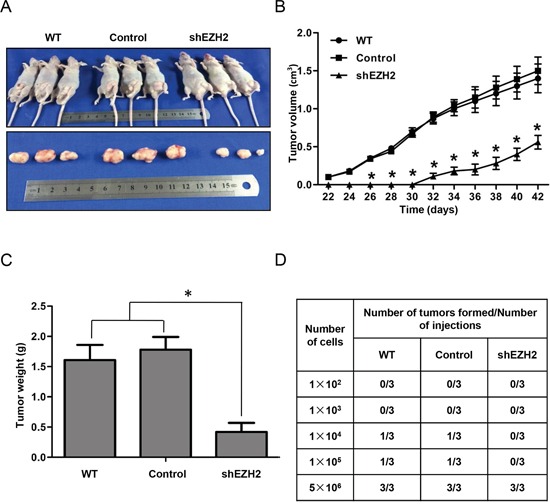
EZH2 knockdown suppressed tumorigenesis and tumor-initiating capacity *in vivo* **A.** Male mice were randomly distributed into three groups, and SW480 cells with or without EZH2 knockdown were subcutaneously inoculated into nude mice. **B.** Xenograft tumor growth was monitored during the experimental period (n = 6). **C.** Xenograft tumor weight is presented in the histogram. **D.** Serial dilutions of cells (100, 1,000, 10,000, 100,000 or 5,000,000 cells) were re-implanted into secondary nude mice. The tumorigenicity ratio of re-implantation is presented in the table (*p<0.05, two-tailed Student's t-tests; the error bars represent the means ± S.D.).

As EZH2 is important for maintaining CCS-like cell properties *in vitro*, we further evaluated whether EZH2 is involved *in vivo* tumorigenicity using a tumor xenograft model. We subcutaneously inoculated 5×10^6^ WT, control or shEZH2 SW480 cells into mice, and primary tumors were allowed to form for 42 days (Figure 5A). The tumors from the shEZH2 group were significantly smaller than those from the WT and control groups (Figure 5B). Consistently, the tumor weight was lower in the shEZH2 group than in the WT and control groups (Figure 5C).

To further demonstrate that EZH2 knockdown reduces tumor-initiating capacity *in vivo*, we performed a functional *in vivo* assay by re-implanting cells from primary tumors into secondary nude mice. This assay is a direct assessment of the tumor-initiating and self-renewal capacities of CCS-like cells [50]. We subcutaneously injected 1×10^2^, 1×10^3^, 1×10^4^, 1×10^5^, or 5×10^6^ tumor cells isolated from primary xenografts of the WT, control or EZH2 knockdown group into secondary nude mice. As shown in Figure 5D, cells from shEZH2-transfected primary tumors showed a 30% reduction in tumorigenesis compared to cells from WT or control-transfected primary tumors. Taken together, these data demonstrate that silencing EZH2 reduced CCS-like cell properties.

### EZH2 knockdown induced CCS-like cell apoptosis

**Figure 6:**
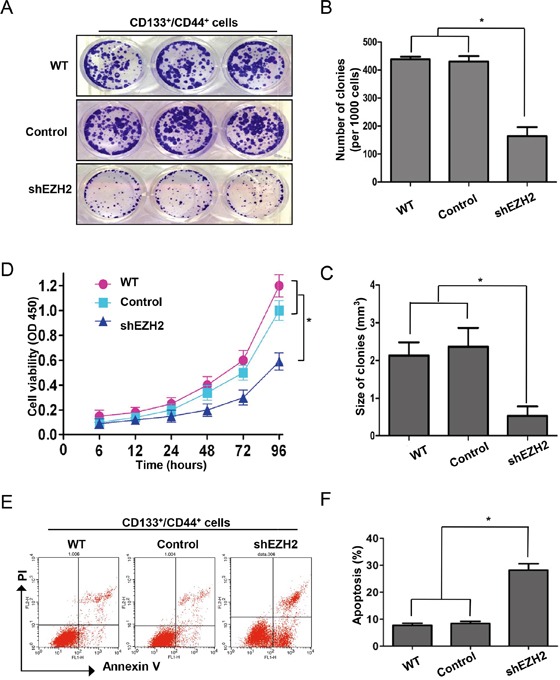
EZH2 knockdown induced CCS-like cell apoptosis **A.** The dissociated CD133^+^/CD44^+^ population of SW480 cells with or without EZH2 knockdown was seeded in 6-cm dishes for 10 days. Representative images of the colonies are presented. **B.** Colony size and **C.** number are presented as the means ± S.D. of three independent experiments. **D.** The viability of the CD133^+^/CD44^+^ population of SW480 cells with or without EZH2 knockdown were assessed via CCK-8 assays at the indicated time points. **E.** Representative results of flow cytometry show the effect of shEZH2 transfection on CD133^+^/CD44^+^ cell apoptosis. **F.** The histogram displays the percentage of apoptotic SW480 cells in the WT, control-transfected and shEZH2-transfected groups from three independent experiments (*p<0.05, two-tailed Student's t-tests; the error bars represent the means ± S.D.).

Previous studies suggested that CS-like cell properties are often suppressed due to apoptosis of CS-like cells [51] or differentiation from CS-like cells into non-CS-like cells [52]. We knocked down EZH2 in CD133^+^/CD44^+^ SW480 cells and performed colony formation assays to evaluate CCS-like cell proliferation (Figure 6A). The results showed that EZH2 knockdown reduced the number (164.0 ± 32.0) (Figure 6B) and size (0.5 ± 0.1 mm^3^) (Figure 6C) of CD133^+^/CD44^+^ cell colonies compared with non-treatment (438.3 ± 9.5 for colony number and 2.1 ± 0.6 mm^3^ for colony size) and control transfection (430.3 ± 19.6 for colony number and 2.3 ± 0.8 mm^3^ for colony size) (p<0.05). Consistently, CCK-8 assays showed that CD133^+^/CD44^+^ SW480 cell viability was significantly decreased by EZH2 knockdown compared with non-treatment and control transfection (p<0.05) (Figure 6D).

To further specify the mechanism by which EZH2 silencing inhibited CD133^+^/CD44^+^ SW480 cell proliferation, we analyzed apoptosis in CD133^+^/CD44^+^ EZH2-silenced SW480 cells via Annexin V and propidium iodide (PI) staining (Figure 6E). As shown in Figure 6F, the apoptosis rate of shEZH2-transfected cells was significantly higher (28.2 ± 2.4%) than that of WT (7.7 ± 0.8%) and control-transfected cells (8.4 ± 0.8%). Thus, EZH2 knockdown reduced the CCS-like cell population by inducing apoptosis.

### EZH2 knockdown inactivated the Wnt/β-catenin signaling pathway by increasing p21^cip1^ expression, leading to G1/S phase arrest

**Figure 7:**
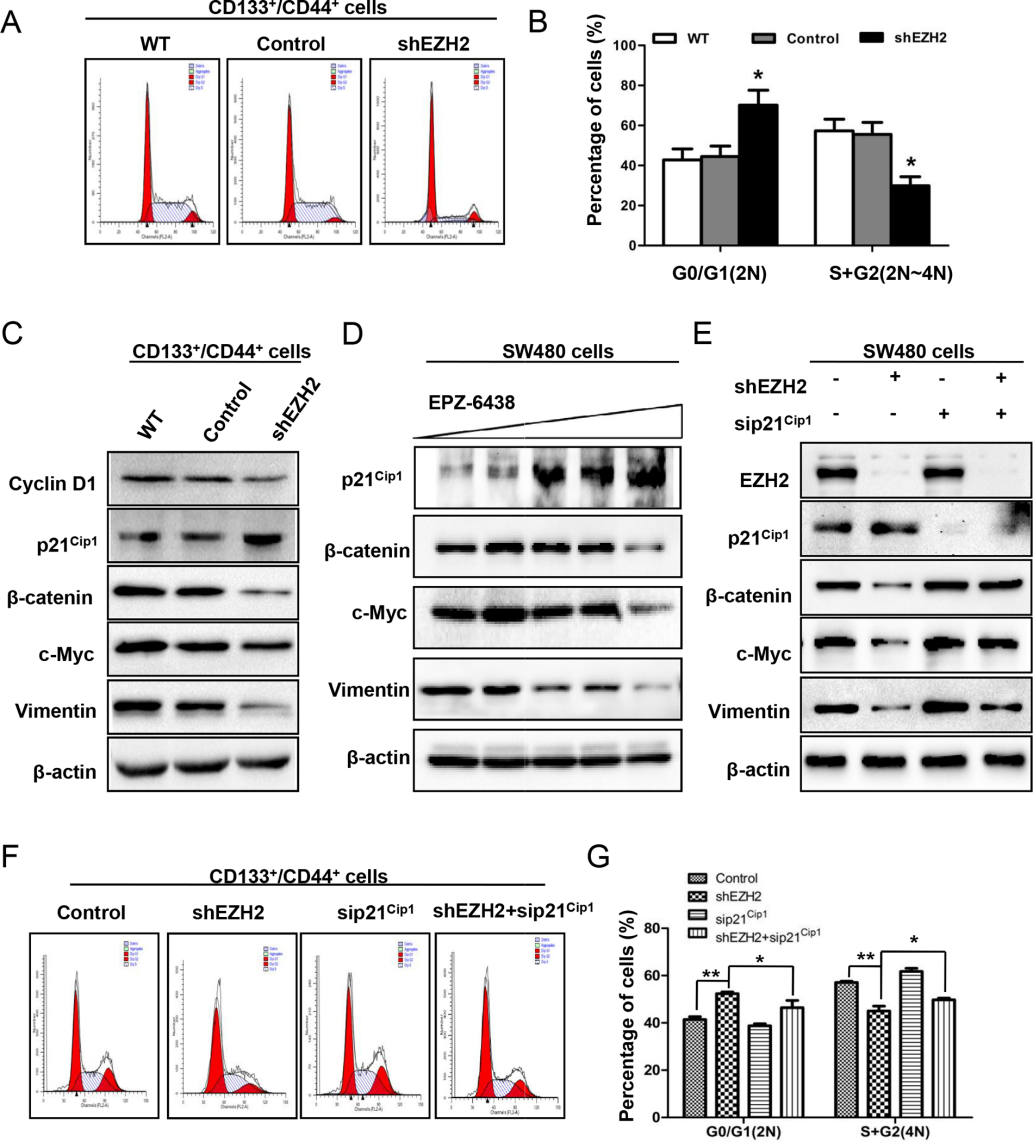
EZH2 knockdown inactivated the Wnt/β-catenin signaling pathway by increasing p21^cip1^ expression, leading to G1/S phase arrest **A.** Cells were fixed and stained with PI. The cell cycle status of the CD133^+^/CD44^+^ population of SW480 cells with or without EZH2 knockdown was analyzed using a FACSCalibur flow cytometer. **B.** The percentage of cells in G0/G1 and S/G2 phase is shown as the means ± S.D. of three independent experiments. **C.** The CD133^+^/CD44^+^ population of SW480 cells with or without EZH2 knockdown was subjected to western blot analysis of cyclin D1 and p21^cip1^ expression. The expression levels of Wnt/β–catenin pathway target genes, including β–catenin, c-Myc, and vimentin, were decreased by EZH2 knockdown. **D.** SW480 cells were incubated with EPZ-6438 at increasing concentrations (0, 2.5, 5, 10, or 20 μM) and subjected to western blot to determine the expression levels of p21^cip1^, β-catenin, c-Myc and vimentin. **E.** The effect of p21^cip1^ knockdown in EZH2-silenced SW480 cells on β–catenin, c-Myc and vimentin expression was detected by western blot. **F.** Representative results of cell cycle analysis of the CD133^+^/CD44^+^ population of SW480 cells in which EZH2 and p21^cip1^ were simultaneously knocked down. **G.** The percentages of cells in G0/G1 and S/G2 phase are shown as the means ± S.D. (*p<0.05, Student's t test).

The cell cycle machinery is involved in the maintenance or suppression of CS-like cell properties [29]. Therefore, we silenced EZH2 in sorted CD133^+^/CD44^+^ SW480 cells and performed cell cycle analysis (Figure 7A). Flow cytometry analysis revealed that the percentage of cells in G0/G1 (2N) phase increased from 42.8 ± 5.5% of WT cells and 44.5 ± 5.2% of control cells to 70.2 ± 7.5% of EZH2-silenced cells (Figure 7B). In contrast, the percentage of cells containing 2N-4N DNA (in the S or G2 phase) decreased from 57.3 ± 5.9% of WT cells and 55.5 ± 6.0% of control cells to 29.9 ± 4.5% of EZH2-silenced cells (Figure 7B). We also analyzed cell cycle marker expression by western blot. In response to EZH2 knockdown, cyclin D1 expression decreased (Figure 7C), which indicated G1/S phase arrest. P21^cip1^ is a cell cycle regulator that can arrest cell cycle progression at the G1/S phase [32]. We found that p21^cip1^ expression was increased following EZH2 knockdown (Figure 7C). Consistent with WT SW480 cells, the CD133^+^/CD44^+^ population showed reduced β-catenin, c-Myc and vimentin expression after knockdown EZH2, indicating that silencing EZH2 inactivated the Wnt/β-catenin pathway (Figure 7C). EPZ-6438 is a specific inhibitor of EZH2 [53]. Similar to shEZH2 transfection, EPZ-6438 treatment increased p21^cip1^ expression and inactivated the Wnt/β-catenin pathway (Figure 7D).

Interestingly, upon siRNA-mediated p21^cip1^ knockdown in EZH2-silenced SW480 cells, the expression levels of β-catenin, c-Myc and vimentin were rescued compared with EZH2 knockdown alone (Figure 7E). Moreover, the cell cycle was analyzed in the CD133^+^/CD44^+^ population of SW480 cells in which EZH2 and p21^cip1^ were simultaneously knocked down (Figure 7F). G1/S arrest was rescued by p21^cip1^ knockdown (Figure 7G). These results established that EZH2 knockdown inactivates the Wnt/β-catenin signaling pathway by increasing p21^cip1^ expression, resulting in G1/S phase arrest.

### EZH2 inhibitor EPZ-6438 delayed tumor growth in a xenograft model

**Figure 8:**
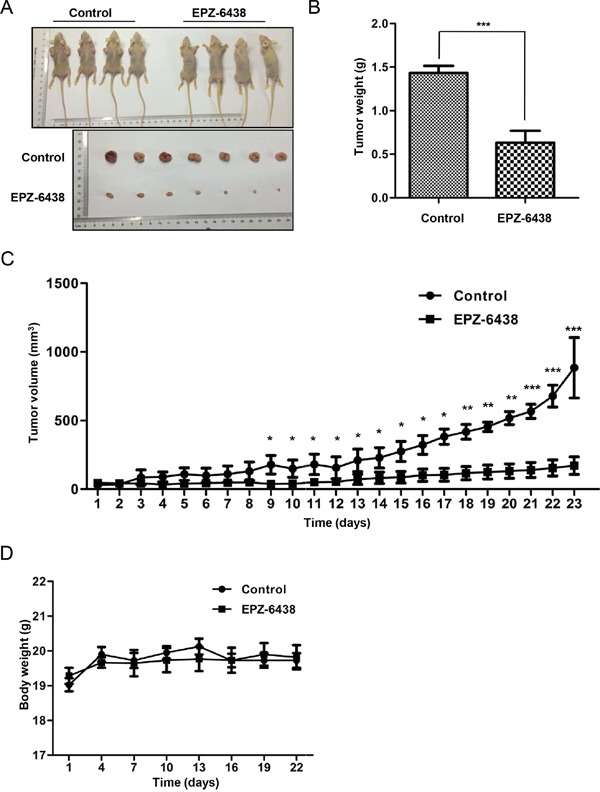
EZH2 inhibitor EPZ-6438 delayed tumor growth in a xenograft model **A.** SW480 cells with or without EZH2 knockdown were inoculated into the right flank of nude mice. The tumors were allowed to develop for 7 days, after which the mice were randomly distributed into two groups, EPZ-6438-treated (50 mg/kg) or vehicle-treated. **B.** Tumor weights of the control and EPZ-6438-treated groups are presented. **C.** The tumor growth rates were monitored during the experimental period (n = 4). **D.** The body weight of the mice was monitored during the experiment (*p<0.05, **p<0.01, and ***p<0.001, Student's t test; the error bars represent the means ± S.D.).

To further explore the clinical application of EZH2 knockdown to inhibit CCS-like cell properties, we used the clinical trial drug EPZ-6438 to perform xenograft experiments. EPZ-6438 is currently in phase I trials in patients with refractory or relapsed B-cell lymphoma [53, 54]. We subcutaneously inoculated SW480 cells into the left flank of nude mice and evaluated tumor growth. After allowing the tumors to develop for 7 days (~100 mm^3^), we randomly allocated the mice to the EPZ-6438 (50 mg/kg) and vehicle control groups (0.5% NaCl + 0.1% Tween-80 in dH_2_O). We administered the drug via oral gavage at the indicated doses once a day for 23 days (Figure 8A). Smaller tumors were observed in the EPZ-6438-treated group (171.9 ± 63.9 mm^3^, p = 0.0009) than in the control group (884.2 ± 220.4 mm^3^) (Figure 8C). The tumor weight was also decreased by EPZ-6438 treatment (Figure 8B). The mice in the EPZ-6438-treated group showed no evident difference in body weight compared to the control mice (Figure 8D). Mouse behaviors, feeding patterns and overall activity were not significantly different between the groups. Collectively, these results suggested that EPZ-6438 inhibits tumorigenicity with favorable a toxicological profile in a xenograft model.

## DISCUSSION

These new data provide compelling evidence that EZH2 promotes CCS-like cell expansion and CRC progression. We further revealed a previously unknown but critical mechanism in which EZH2 activates the Wnt/β-catenin signaling pathway by decreasing p21^cip1^ expression, which suppresses G1/S phase arrest (Figure 7A-7F). These findings demonstrate that inhibiting EZH2 and its downstream activation of pathways, such as inactivating p21^cip1^-Wnt/β-catenin signaling pathway was effective in eliminating CCS-like cells and reducing CRC progression.

Previous studies showed that EZH2 is a critical predictor of cancer prognosis. EZH2 has high prognostic value for prostate cancer and potentially contributes to the development of a subtype of genetically unstable and particularly aggressive prostate cancers [55]. High EZH2 expression is associated with an undifferentiated cellular state in nasopharyngeal carcinoma patient samples [56]. In contrast, inhibiting EZH2 reduces tumor invasion and angiogenesis [57]. In the present study, we determined that EZH2 might be an oncogene that performs an important function in CRC tumor development. Moreover, we demonstrated that EHZ2 is indispensable to CCS-like cells. CCS-like cells are considered as the origin of cancer. Indeed, as shown in Figure 1, the immunohistochemistry results showed that normal tissue rarely expressed EZH2. Therefore, EHZ2 not only serves as a predictor of cancer prognosis but also functions as a tumorigenic factor in CRC. Thus, interfering with EZH2 expression or activation is a promising strategy for CRC therapy or for preventing CRC tumorigenesis.

CS-like cells dynamically interchange between dormant (G0 phase) and cycling phases [58, 59]. A rare cell subset comprising 0.01–1% of the total CS-like cell population was sufficient to induce carcinoma when transplanted into immunodeficient mice [60, 61]. This finding suggested that cycling CS-like cells are a target for eradicating cancer [62]. Indeed, accumulating studies have highlighted that arresting the cell cycle in CS-like cells decreases tumorigenesis [63–65]. For example, the E3 ligase Skp2, a critical factor in cell-cycle progression, restricted cancer progression and metastasis [63]. Flubendazole treatment arrests cells in the G2/M phase, suppresses CS-like cell migration, and induces breast cancer differentiation [65]. Here, we identified a novel function of EZH2 based on the finding that silencing EZH2 arrested CCS-like cells in the G1/S phase (Figure 7A-7B). However, agents such as adriamycin arrest the cell cycle and induce a quiescent state rather than depleting cancer cells, and this effect has been reported to contribute to cancer recurrence [66, 67]. CS-like cells may have an inherent ability to escape depletion resulting from cell cycle arrest, as these cells exhibit defects in cell cycle checkpoints [68–70]. Surprisingly, EZH2 knockdown preferentially inhibits CCS-like cells by inducing CCS-like cell apoptosis (Figure 6E-6F). Therefore, we conclude that cell cycle arrest is a pivotal contributor to the eradication of CS-like cells to cure cancer via the regulation of EZH2 expression.

The mechanism by which EZH2 contributes to the maintenance of CS-like cell properties in CRC is not clearly understood. Here, we suggest that suppressing EZH2 expression caused an increase in p21^cip1^ expression, which was associated with the maintenance of CS-like cell properties (Figure 7C-7D). H3K27 is trimethylated at the p21^cip1^ promoter upon direct binding to EZH2 [71]. Indeed, loss of p21^cip1^ is associated with CS-like cell properties, epithelial-mesenchymal transition and carcinoma progression [72, 73]. Inhibiting H3K27 methylation at the p21^cip1^ promoter increases p21^cip1^ expression and elevates the stemness properties of embryonic stem cells [74]. Thus, the decrease of p21^cip1^ expression caused by EZH2 may be a consequence of EZH2 epigenetic silencing.

The critical mechanism by which p21^cip1^ suppresses CS-like properties is controversial [36–39]. Here, we demonstrated a critical mechanism for the inhibition of Wnt/β-catenin pathway activity by p21^cip1^ in CRC. β-catenin expression was rescued by p21^cip1^ knockdown after silencing EZH2 (Figure 7E). This effect was accompanied by an increase in c-Myc and vimentin expression, reflecting Wnt/β-catenin pathway activation (Figure 7E). Additionally, p21^cip1^ inhibits the cell cycle by phosphorylating critical substrates such as cyclin-dependent kinase 4 (CDK4) [33, 34]. Moreover, in canonical Wnt signaling, the Wnt pathway is activated by increasing β-catenin stability via β-catenin phosphorylation at different sites [75]. Phosphorylation at serine residues 552 and 675 prevents β-catenin degradation and increases β-catenin transcriptional activity [76–79]. Conversely, β-catenin is ubiquitinated and degraded by casein kinase 1 at serine 45 and by glycogen synthase kinase 3 (GSK3) at threonine 41, serine 37, and serine 33 [78, 79]. Therefore, these results may establish a mechanism by which EZH2 promotes CS-like properties by inhibiting p21^cip1^ expression via the enhancement of H3K27 methylation at the p21^cip1^ promoter, thereby altering β-catenin phosphorylation.

EPZ-6438 has generally been used to treat lymphoma patients [53]. Consistent with its effects on B-cell lymphoma, EPZ-6438 suppressed the growth of CRC xenografts (Figure 8). Previous studies revealed that heterogeneity in gene expression may predict the effectiveness of individualized treatment of CRC patients [80]. We suggest a mechanism that EZH2 suppresses p21^cip1^ expression to maintain CCS-like cell properties (Figure 7). Knocking down p21^cip1^ in EZH2-silenced cells resulted in stronger Wnt/β-catenin signaling inactivation compared with EZH2 knockdown alone (Figure 7E). Loss of p21^cip1^ in tumors has been observed in a portion of CRC patients [81]. Thus, our results suggest that EZH2 effectively eliminates CCS-like cells and improves CRC patient prognosis in a p21^cip1^-dependent manner. Therefore, EPZ-6438 holds clinical potential for the selective treatment of CRC patients expressing p21^cip1^. Our findings provide individualized selection criteria for the application of EPZ-6438 to CRC therapy.

In summary, we demonstrated that EZH2 performs a pivotal and essential function in maintaining CCS-like properties and that repression of p21^cip1^ by EZH2 is a novel regulatory mechanism of Wnt/β-catenin signaling inactivation. These data suggest a promising molecular target for eradicating CCS-like cells and open new avenues for targeted CRC therapy.

## MATERIALS AND METHODS

### Patients and tissue specimens

Paraffin-embedded CRC specimens and fresh frozen CRC tissues (n = 81) (Table 1) were obtained from the Southwest Hospital of the Third Military Medical University, Chongqing, China. The specimens included 3 T1 status CRC tissues, 6 T2 tissues, 63 T3 tissues, and 9 T4 tissues. All samples were confirmed by pathological examination, and staging was performed according to the 1997 CRC staging system of the UICC. Among the 81 CRC samples, 41 were from male patients and 40 were from female patients, with ages ranging from 24 to 90 years (median, 70 years). Informed consent was obtained from all patients, and this study was approved by the ethics committee of Southwest Hospital of the Third Military Medical University.

### Statistical analysis of microarray data

Gene expression data from TCGA database were obtained at https://genome-cancer.ucsc.edu. Pearson's correlation coefficients were used to determine the P values for co-expression.

### Cell culture

Human CRC cell lines (SW480, SW620, and LoVo) were purchased from American Type Culture Collection (Manassas, VA, USA). The SW480 and SW620 cells were maintained in Leibovitz's L-15 Medium (Invitrogen Corp., USA) supplemented with 10% fetal bovine serum (Invitrogen Corp., USA). The LoVo cells were maintained in DMEM (Invitrogen Corp., USA) supplemented with 10% fetal bovine serum (Invitrogen Corp., USA). All cells were incubated at 37°C in a humidified incubator containing 5% CO_2_.

### CCK-8 assay

After transfection with a scramble sequence or shEZH2, SW480 and SW620 cells were seeded in 96-well plates (5×10^3^ cells per well) at 100 μl of cell suspension per well. After transfection with a scramble sequence or EZH2, LoVo cells were also seeded in 96-well plates. After culturing for 0, 24, 48, and 72 h, 10 μl of CCK-8 solution (Invitrogen Corp., USA) was added to each well, and the cells were incubated for an additional 2 h at 37°C. The absorbance was measured at 450 nm using a SpectraMax M5 plate reader (Molecular Devices, Sunnyvale, CA, USA). The assay was conducted in six replicate wells for each sample, and three parallel experiments were performed.

### Colony formation assay

Dissociated untreated, control-transfected or shEZH2-transfected SW480 or SW620 cells were seeded in 6-cm dishes at a density of 1,000 cells per dish. After 15 days in culture, the colonies were fixed in 4% paraformaldehyde at 4°C and stained with 0.4% crystal violet for 10 min at room temperature. Data were acquired from three independent experiments, and the number of colonies was counted.

### Western blot analysis

After transfection with the scramble or shEZH2 plasmid, SW480 and SW620 cells were harvested and lysed in RIPA buffer. Protein concentrations were determined via the Bradford assay. Then, cell lysates (20 μg) were separated via SDS-PAGE and transferred to nitrocellulose membrane (Merck Millipore). The membranes were blocked in 3% skim milk (Becton Dickinson) and incubated with the indicated antibodies, including antibodies against CK20 (Cell Signaling Technology, 1:1000), Nanog (Cell Signaling Technology, 1:1000), Sox2 (Cell Signaling Technology, 1:1000), EZH2 (Cell Signaling Technology, 1:1000), β-catenin (Cell Signaling Technology, 1:5000), vimentin (Cell Signaling Technology, 1:1000), cyclin D1 (Cell Signaling Technology, 1:1000), and p21^cip1^ (Cell Signaling Technology, 1:1000). The membranes were subsequently incubated with the appropriate secondary antibodies (Santa Cruz Biotechnology, Dallas, TX, USA). Specific antibody–antigen complexes were detected using the SuperSignal West Femto Detection Kit (Pierce, Rockford, IL, USA). The membranes were scanned using a ChemiDoc CCD camera system (BioRad, USA). The Western blots shown are representative of at least three independent experiments. β-actin (Cell Signaling Technology, 1:5000) was used as the loading control.

### Mammosphere formation assay

Dissociated single cells (1,000 per well) were seeded in 6-well ultra-low-attachment plates (Corning Costar). The cells were cultured in serum-free DMEM/F-12 (Invitrogen Corp., USA) supplemented with 20 ng/ml epithelial growth factor (EGF, Sigma Aldrich, USA), 20 ng/ml basic fibroblast growth factor (bFGF, PeproTech) and B27 (BD Biosciences, USA) for 9 days. All spheres were photographed under an inverted microscope (10×, Olympus). The number and diameter of spheres were calculated using Image-Pro Plus 6.0 software (Olympus).

### Flow cytometry analysis

For CD133^+^/CD44^+^ subpopulation detection, SW480 cells were harvested and washed twice with PBS via centrifugation (1,000 rpm, 5 min). The collected cells were incubated with APC-conjugated anti-CD133 (Miltenyi Biotec, DE) and PE-conjugated anti-CD44 antibodies (BD Biosciences, USA) or isotype control antibodies according to the manufacturer's instructions for 30 min at 4°C while protected from light. The cells were then suspended in 1 ml of PBS and subjected to flow cytometry analysis (Beckman, USA). Side-scatter and forward-scatter profiles were used to eliminate cell doublets [82]. The statistical results of three independent experiments are presented.

For isolation of the CD133^+^/CD44^+^ subpopulation from SW480 cells, the above steps were followed, but the cells were sorted via fluorescence-activated cell sorting (FACS).

For the detection of apoptosis, cells were harvested and dissociated in PBS. The samples were stained with Annexin V-FITC and PI and analyzed by flow cytometry using FlowJo software (Tree Star, Ashland, OR, USA).

For cell cycle analysis, DNA content was examined by PI staining. Briefly, cells were seeded in 6-cm dishes, and both adherent and floating cells were collected for analysis. The cells were fixed in 70% cold ethanol, stained with 1 mg/ml PI and analyzed using a FACSCalibur flow cytometer (FACSCan, Becton Dickinson, San Jose, CA). The fluorescence profiles represent the DNA contents of PI-positive cells.

### Immunohistochemical staining and statistical analysis

Paraffin-embedded tissue blocks were sectioned, deparaffinized in xylene and rehydrated for immunohistochemical staining [83]. Antigen retrieval was performed using sodium citrate. The sections were then incubated in H_2_O_2_ (3%) for 10 min, blocked in 1% bovine serum albumin for 60 min and incubated with an anti-EZH2 antibody (Cell Signaling Technology, 1:400) at 4°C overnight. After incubation with a secondary antibody for 60 min, the specimens were incubated with H_2_O_2_-diaminobenzidine until the desired staining intensity was observed. The sections were counterstained with hematoxylin, dehydrated and mounted. Immunohistochemical staining was evaluated in accordance with the immunoreactive score (IRS), in which IRS = staining intensity (SI) X percentage of positive cells (PP). SI was scored as follows: negative SI = 0; weak SI = 1; moderate SI = 2; and strong SI = 3. Similarly, PP was scored as follows: negative PP = 0; 10% PP = 1; 11–50% PP = 2; 51–80% PP = 3; and >80% PP = 4. All specimens used for immunohistochemical staining were evaluated and scored by at least two independent pathologists.

### Plasmid construction and transfection

Lentiviral particles for EZH2 were purchased from Neuron Biotech Co., Ltd. (Shanghai, China). Cells were incubated with lentivirus and polybrene for 12-16 h, after which the medium was changed. Seven days after transfection, the cells were incubated in selection medium containing 0.5 mg/ml G418 (Life Technologies, Inc., Carlsbad, CA, USA). After 14 days of selection, individual G418-resistant colonies were subcloned. Protein expression was analyzed by immunoblotting. The target sequence for p21^cip1^ siRNA was 5′-ACAAGGGUACAAGACAGUGAC-3′ (Sangon Biotech, Shanghai), and negative control siRNA sequence was 5′-CCACUACCUGAGCACCCAGUU-3′ (Sangon Biotech, Shanghai). siRNA oligonucleotides were diluted in DEPC water to 20 μmol/L. Opti-MEM (Invitrogen Corp., USA) containing siRNA oligonucleotides (10 μl) was transfected into SW480 cells using Lipofectamine 2000 (#11668-019, Invitrogen Corp., USA) according to the manufacturer's protocol.

### RT-qPCR

Total RNA was extracted using TRIzol reagent (Invitrogen Corp., USA) and was employed to generate cDNA using Superscript III RT (Invitrogen Corp., USA) and an oligo(dT) primer. Real-time RT-PCR was performed using Platinum SYBR Green qPCR SuperMix (Invitrogen Corp., USA) according to the manufacturer's protocol. GAPDH was used as the internal control. The primers used are as follows: GAPDH: forward, 5′-GGAGTCCACTGGCGTCTT-3′, reverse, 5′-TCTTGAGGCTGTTGTCATACTT-3′; EZH2: forward, 5′-GGCACAGCAGAAGAACTA-3′, reverse, 5′-AACATCGCCTACAGAAAA-3′.

### Animal model

For re-implantation assays, SW480 cells (untreated, control-transfected or shEZH2-transfected) were subcutaneously implanted into 4-6-week-old nude mice (n = 6). The tumor volume (a×b^2^/2; a represents the greatest diameter, and b represents the diameter perpendicular to a) was measured with calipers twice a week. After 42 days, the mice were euthanized, and the xenograft tumors were immediately dissected. After mincing, the xenograft tissue was trypsinized in 0.5% collagenase I and then cultured in Leibovitz's L-15 Medium (Invitrogen Corp., USA) supplemented with 10% fetal bovine serum (Invitrogen Corp., USA). After 12-16 h, the adherent cells were harvested and re-implanted into secondary nude mice at the appropriate density.

For the drug treatment model, SW480 cells (5×10^6^) were subcutaneously implanted into 4-6-week-old nude mice (n = 6). After 7 days, all mice that developed tumors ~100 mm^3^ in volume were randomly allocated to receive either EPZ-6438 or vehicle (0.5% NaCMC + 0.1% Tween-80 in water) at 50 mg/kg daily via oral gavage [53]. The tumor volume (a×b^2^/2; a represents the greatest diameter, and b represents the diameter perpendicular to a) was measured every day using calipers. Other indicators of general health were monitored. After 23 days, the mice were euthanized, and the xenograft tumors were immediately dissected, weighed, stored and fixed. All of our animal studies were approved by the Institutional Animal Care and Use Committee of the Third Military Medical University.

### Statistical analysis

Each experiment was performed in triplicate and was repeated at least three times. Unless otherwise indicated, the data are presented as the means ± S.D. of three independent experiments. Statistics were calculated using SPSS software (version 16.0). Differences in a given variable between groups were assessed using two-tailed Student's t-tests, Fisher's exact test or Pearson's χ2 test. A p-value of less than 0.05 was considered statistically significant (*p<0.05, **p<0.01, ***p<0.001). For cancer survival analysis, Kaplan–Meier survival curves of overall survival were used.

## SUPPLEMENTARY FIGURES



## Abbreviations

CS-like cell: cancer stem-like cell
CCS-like cell: colorectal cancer stem-like cell
CRC: colorectal cancer
EZH2: polycomb group protein enhancer of zeste homologue 2
shRNA: short hairpin RNA
WT: wild type
