# Supplementary material for: EZH2 promotes colorectal cancer stem-like cell expansion by activating p21cip1-Wnt/β-catenin signaling
Source: Oncotarget. 2016 May 9;7(27):41540–58. doi: 10.18632/oncotarget.9236 (PMC5173077; doi:10.18632/oncotarget.9236)
Supplement: Supplementary file 1 [file oncotarget-07-41540-s001.pdf]

EZH2 promotes colorectal cancer stem-like cell expansion by activating p21^{cip1}-Wnt/β-catenin signaling

SUPPLEMENTARY FIGURES

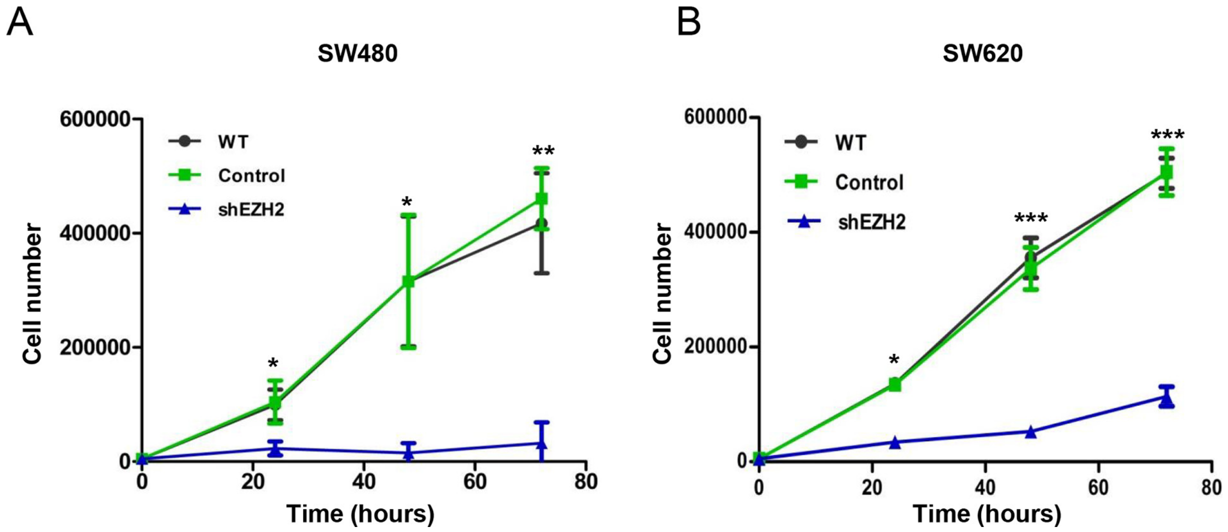

Supplementary Figure S1: EZH2 promoted proliferation and clonogenicity of CRC cells. EZH2 was knocked down in **A.** SW480 and **B.** SW620. After 24, 48 and 72 hr of incubation, cell proliferation was measured by cell counting assay.

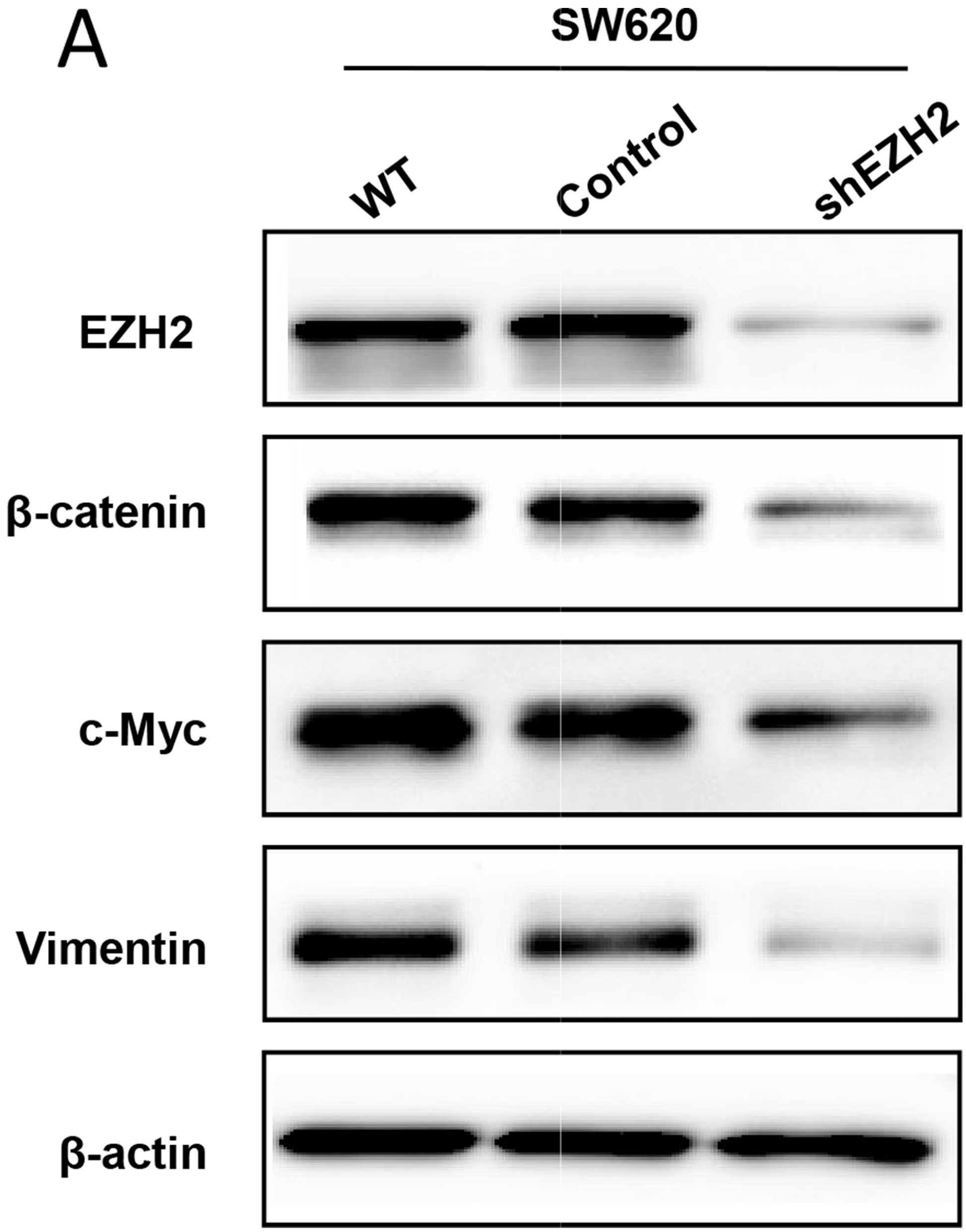

Supplementary Figure S2: Silencing EZH2 expression inactived Wnt/β-catenin signaling pathway in SW620 cell line. **A.** The expression of β–catenin, vimentin and c-myc were detected by western blot after knockdown EZH2 in SW620 cells.

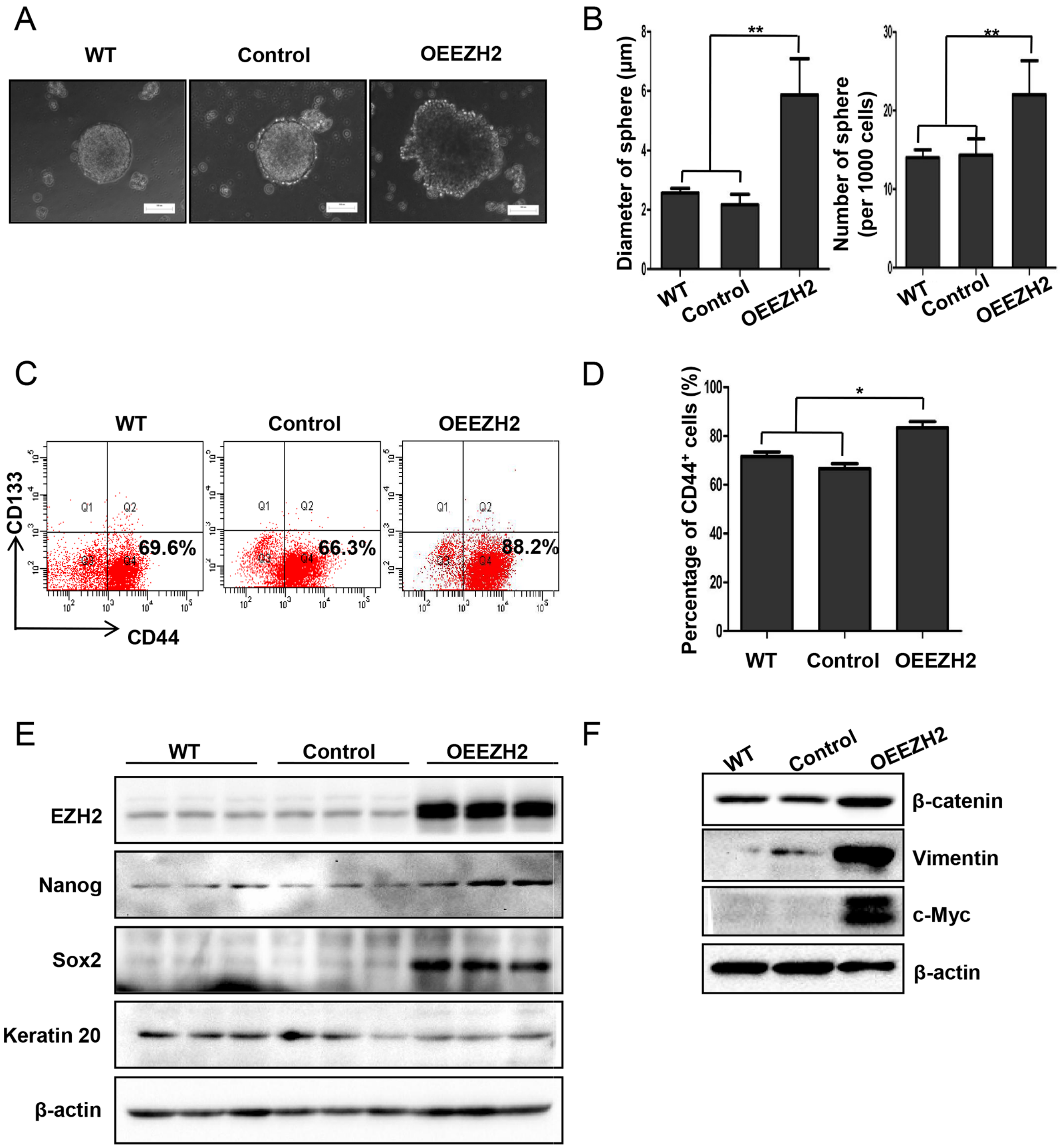

Supplementary Figure S3: Overexpression EZH2 promoted stemness of LoVo cell line. A. Wild type (WT), scramble OERNA (Control) and OEEZH2 of LoVo cells were plated in low-serum non-adherent culture conditions. Images were obtained by microscopy at 10× magnification and were representative of all mammospheres formed (Scale bar = 100 μm). **B.** The size and number of LoVo cell spheres were analyzed. Data were presented as the mean ± S.D. of three independent experiments. **C.** $CD133^+/CD44^+$ population was analyzed by flow cytometry analysis in LoVo cells with or without EZH2 overexpression. **D.** The statistical results of three independent experiments were presented. **E.** After EZH2 knockdown, the expression of EZH2, Nanog, Sox2, and Keratin 20 was examined by western blot. **F.** The expression of β–catenin, vimentin and c-myc were detected by western blot after overexpression EZH2 in LoVo cells.
